# Association of acupuncture combined with therapeutic massage with clinical outcomes in cervical radiculopathy: A retrospective cohort study

**DOI:** 10.1097/MD.0000000000049993

**Published:** 2026-08-07

**Authors:** Liqing Qi, Hong Liang, Zhan Wen Li

**Affiliations:** a Third Department of Orthopedics and Traumatology, Hebei Provincial Hospital of Traditional Chinese Medicine, Shijiazhuang, Hebei, P.R. China; b PuDong New District, Huamu Community Health Service Center, Shanghai, P.R. China; c Third Ward, Department of Tuina, Yulin Municipal Hospital of Traditional Chinese Medicine, Yulin, Guangxi Zhuang Autonomous Region, P.R. China.

**Keywords:** acupuncture, cervical radiculopathy, cervical spondylotic radiculopathy, neck disability index, therapeutic massage, Tuina

## Abstract

Cervical radiculopathy is associated with neck pain radiating to the upper-limb, sensory symptoms, restricted cervical mobility, and functional disability. Acupuncture and therapeutic massage are commonly used conservative treatments, but real-world comparative evidence evaluating their combined use remains less well characterized. This study aimed to evaluate the association between acupuncture combined with therapeutic massage and clinical outcomes in patients with cervical radiculopathy. This retrospective cohort study included 126 adults with clinically diagnosed cervical radiculopathy treated between January 2021 and December 2024. Patients treated with acupuncture alone were compared with those treated with acupuncture-plus therapeutic massage. The primary outcome was change in Neck Disability Index (NDI) score from baseline to the end-of-treatment. Secondary outcomes included visual analog scale pain score, cervical range of motion, global clinical response, 3-month recurrence, medication reduction, and adverse events. Adjusted regression and propensity-score sensitivity analyses were used to address measured baseline differences. Sixty patients received acupuncture alone and 66 received acupuncture-plus therapeutic massage. Baseline characteristics were similar between groups. Compared with acupuncture alone, combined-therapy was associated with greater improvement in NDI score (−23.4 vs −16.2 points), visual analog scale pain score (−3.4 vs −2.4 points), cervical range of motion (+19.6 vs +12.5 degrees), and marked or partial clinical response (83.3% vs 65.0%). After adjustment, combined-therapy remained associated with greater NDI improvement, greater pain reduction, and a higher clinical response rate; recurrence was numerically lower in the combined-therapy group. No serious treatment-related adverse events were documented. Acupuncture combined with therapeutic massage was associated with greater short-term improvement in pain, disability, cervical mobility, and clinical response than acupuncture alone. Because this was a retrospective observational study, prospective multicenter studies with standardized treatment protocols and longer follow-up are warranted.

## 1. Introduction

Cervical radiculopathy is characterized by neck pain radiating to the upper-limb due to irritation or compression of one or more cervical nerve roots.^[1]^ Degenerative foraminal stenosis and disc herniation are common mechanisms, and the C6 and C7 nerve-root distributions are frequently involved in clinical cohorts.^[1,2]^

Cervical radiculopathy is clinically important because it can cause persistent pain, sleep disturbance, restricted cervical range of motion (ROM), impaired upper-limb function, reduced work productivity, and recurrent use of analgesic, rehabilitation, or interventional services.^[2]^ Conservative treatment varies across settings and may include education, medication, traction, exercise, mobilization, acupuncture, massage, or multimodal care, with many patients improving without surgery.^[2]^

Acupuncture is commonly used for cervical radicular pain and may reduce symptoms through peripheral and central neuromodulatory mechanisms, including local pain modulation and anti-inflammatory effects.^[3]^ Therapeutic massage, including Tuina-based techniques, may reduce soft-tissue guarding, improve cervical mobility, and complement active rehabilitation, although systematic reviews have emphasized heterogeneity and limited certainty of evidence for cervical radiculopathy.^[4,5]^

Although acupuncture and Tuina/massage have been studied previously, fewer real-world cohort studies have compared acupuncture alone with acupuncture-plus therapeutic massage while assessing disability, pain, cervical motion, recurrence, and safety in the same cohort.^[4–6]^ Existing records can help answer this question because outpatient rehabilitation and traditional medicine clinics routinely document diagnosis, symptom duration, suspected level, imaging findings, treatment sessions, visual analog scale (VAS) pain scores, Neck Disability Index (NDI) disability scores, cervical ROM, clinical response, adverse events, and follow-up contacts.^[7–9]^

The aim of this retrospective cohort study was to evaluate the association between acupuncture combined with therapeutic massage and clinical outcomes in patients with cervical radiculopathy. The primary objective was to compare change in NDI score between the acupuncture-plus-massage group and the acupuncture alone group. Secondary objectives were to compare pain reduction, cervical range-of-motion improvement, global clinical response, recurrence, safety, and treatment-response patterns by symptom duration, baseline disability, and dominant pathoanatomic pattern using previously collected clinical data.^[7–10]^

## 2. Methods

### 2.1. Study design and setting

This retrospective observational cohort study was reported according to Strengthening the Reporting of Observational Studies in Epidemiology principles for observational research.^[7]^ The setting was an outpatient rehabilitation and traditional medicine service that provided conservative care to adults with clinically diagnosed cervical radiculopathy.^[1,2]^

**Figure 1.**
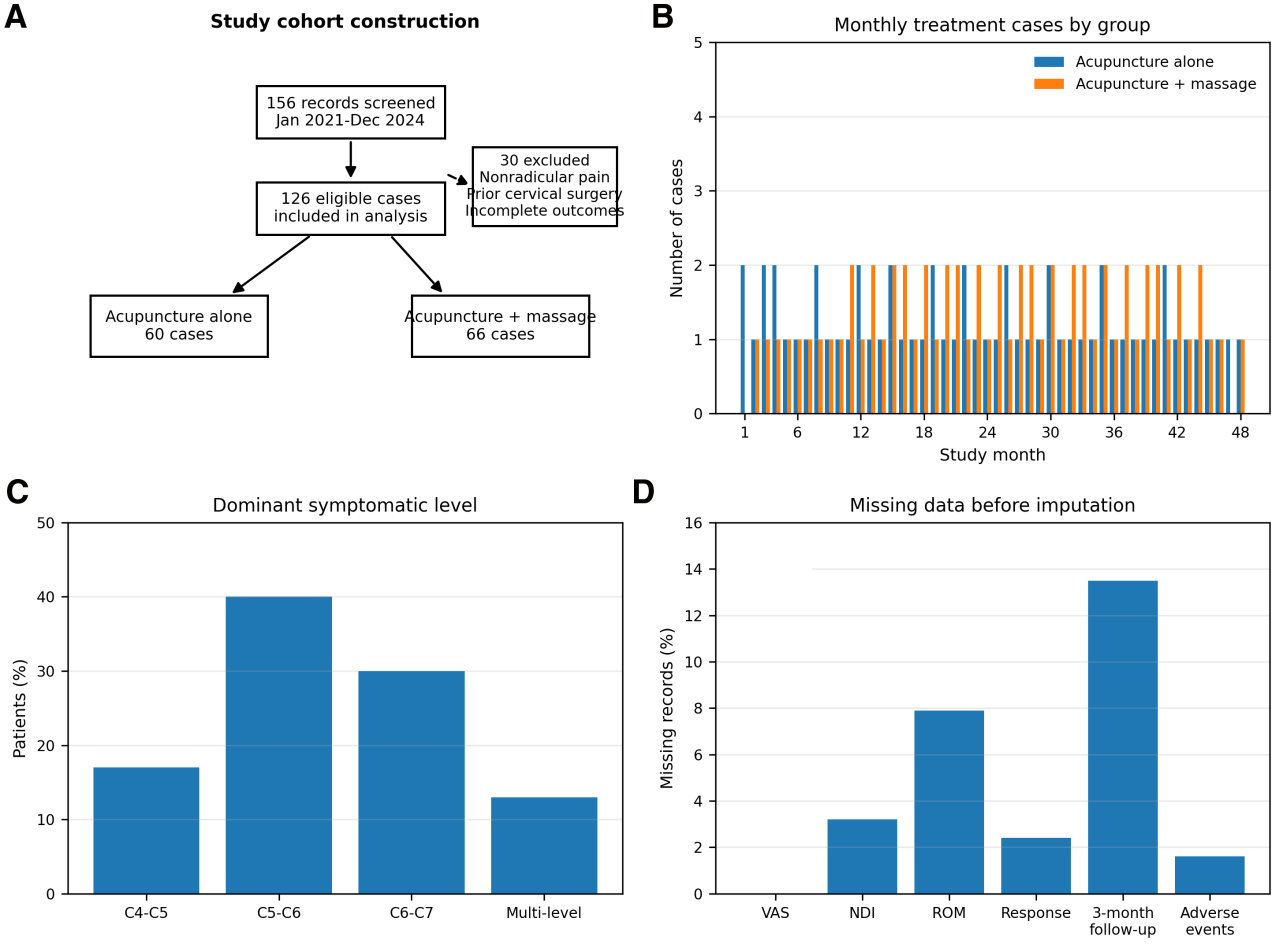
Cohort assembly, treatment-volume pattern, symptomatic level, and missingness. (A) shows the retrospective cohort flow; (B) shows monthly treatment cases by group; (C) shows dominant symptomatic level distribution; (D) shows missing data before imputation.

Records from January 1, 2021, through December 31, 2024, were screened. After prespecified eligibility criteria were applied, 126 patients were included in the analytic cohort, including 60 patients treated with acupuncture alone and 66 patients treated with acupuncture combined with therapeutic massage (Fig. 1A).

A retrospective consecutive sampling method was used. All available clinical records of adult patients treated for clinically diagnosed cervical radiculopathy between January 1, 2021, and December 31, 2024, were screened. Patients who met the predefined eligibility criteria were included in the final analytic cohort.

### 2.2. Ethical review and consent

This retrospective cohort study was reviewed and approved by the Ethics Committee of Yulin Municipal Hospital of Traditional Chinese Medicine. Because the study used previously collected clinical records and did not involve direct patient contact or any additional intervention, the requirement for written informed consent was waived by the ethics committee. Patient data were reviewed and analyzed anonymously to protect patient confidentiality.

### 2.3. Eligibility criteria

Patients were eligible if they were 18 to 80 years old, had neck pain with radiating arm pain or paresthesias consistent with cervical radiculopathy, had documentation of a nerve-root distribution, received at least eight conservative treatment sessions, and had baseline and end-of-treatment NDI or VAS scores available.^[1,2]^

Patients were excluded if symptoms were nonradicular, myelopathy was suspected, prior cervical surgery was documented, or the record indicated acute fracture, tumor, infection, inflammatory arthropathy, insufficient outcome documentation, inability to complete treatment, or a contraindication to safe needling (Fig. 1A).^[1,2,7]^

### 2.4. Exposure definition

The exposure of interest was combined acupuncture and therapeutic massage. Acupuncture sessions used cervical and upper-limb points selected according to the documented symptom distribution. Massage consisted of supervised therapeutic soft-tissue manipulation, kneading, pressing, mobilization-compatible techniques, and relaxation of cervical and scapular muscles.^[4,5]^

The comparison group received acupuncture without documented therapeutic massage during the same treatment course. Education, posture advice, home exercises, and non-opioid analgesics could be provided to either group according to conservative management principles.^[2]^

### 2.5. Data sources and variables

Data were obtained from registration records, clinician notes, acupuncture and massage treatment logs, pain assessment sheets, NDI forms, cervical range-of-motion records, imaging reports, medication lists, adverse-event notes, and 3-month follow-up documentation.^[7–9]^

Extracted variables included age, sex, body mass index, symptom duration, dominant symptom pattern, suspected symptomatic level, imaging pattern, baseline VAS neck-arm pain, baseline NDI score, baseline cervical ROM, treatment group, number of sessions completed, home exercise advice, analgesic use, follow-up attendance, recurrence, and adverse events.^[7–10]^

The 2 treatment groups were compared at 3 assessment points. Baseline comparisons were performed before treatment initiation and included demographic characteristics, symptom duration, imaging pattern, baseline VAS pain score, baseline NDI score, cervical ROM, and analgesic use. End-of-treatment comparisons were performed after completion of the treatment course and included changes in NDI score, VAS pain score, cervical ROM, arm numbness score, medication reduction, and global clinical response. Follow-up comparisons were performed at 3 months after treatment and included recurrence of radicular symptoms and documented adverse events.

### 2.6. Outcomes

The primary outcome was change in NDI score from baseline to the end of the treatment course. The NDI was selected because it is widely used to quantify self-reported neck disability and has been evaluated in neck pain and cervical radiculopathy populations.^[8–10]^

Secondary outcomes included change in VAS pain score, change in active cervical ROM, global clinical response at the end of treatment, recurrence of radicular symptoms at 3 months, medication reduction, and documented adverse events. Global clinical response was classified as marked response, partial response, or minimal/no response according to recorded improvement in pain and disability.^[8–11]^

### 2.7. Bias control and missing data

Consecutive record screening, explicit eligibility criteria, and transparent reporting of cohort flow were used to reduce selection bias (Fig. 1A).^[7]^ Confounding by indication was anticipated because physicians may have recommended combined-therapy based on muscle spasm, symptom duration, patient preference, ability to attend multiple visits, or perceived likelihood of response.^[7,12]^ Potential confounding variables included age, sex, symptom duration, baseline VAS pain score, baseline NDI score, imaging pattern, baseline analgesic use, and number of treatment visits. These variables were controlled using baseline group comparisons, multivariable adjusted regression models, and propensity-score sensitivity analysis.

Baseline comparability was summarized in Table 1. Adjusted models included age, sex, symptom duration, baseline NDI, baseline VAS pain, imaging pattern, baseline analgesic use, and number of visits. Missing data were summarized by outcome domain (Fig. 1D). Complete-case analysis was the primary approach, and multiple-imputation sensitivity analysis was performed for outcomes with more than 5% missingness.^[12,13]^ Three-month follow-up data were available for 109 of 126 patients (86.5%). Follow-up availability was 83.3% in the acupuncture alone group and 89.4% in the acupuncture-plus-massage group, indicating limited follow-up attrition without a marked imbalance between groups.

**Table 1:** Baseline characteristics stratified by treatment exposure.

Characteristic	Overall (n = 126)	Acupuncture alone (n = 60)	Acupuncture + massage (n = 66)	*P*-value
Age, yr, mean (SD)	50.8 (10.6)	51.2 (10.8)	50.4 (10.5)	.67
Female sex, n (%)	68 (54.0)	32 (53.3)	36 (54.5)	.89
Symptom duration, wk, median (IQR)	6.5 (3.0–13.0)	7.0 (3.0–14.0)	6.0 (3.0–12.0)	.48
Symptoms <3 mo, n (%)	88 (69.8)	40 (66.7)	48 (72.7)	.46
Disc herniation pattern, n (%)	58 (46.0)	27 (45.0)	31 (47.0)	.82
Spondylotic foraminal stenosis, n (%)	52 (41.3)	25 (41.7)	27 (40.9)	.93
Dominant C5-C6 or C6-C7 level, n (%)	88 (69.8)	41 (68.3)	47 (71.2)	.72
Baseline VAS pain score, mean (SD)	6.8 (1.2)	6.9 (1.2)	6.8 (1.1)	.64
Baseline NDI score, mean (SD)	41.7 (10.9)	42.1 (10.8)	41.3 (11.1)	.68
Baseline cervical ROM, degrees, mean (SD)	142.6 (26.8)	143.4 (27.1)	141.9 (26.7)	.76
Analgesic use at baseline, n (%)	64 (50.8)	32 (53.3)	32 (48.5)	.59

IQR *=* interquartile range, NDI *=* Neck Disability Index, ROM *=* range of motion, SD *=* standard deviation, VAS *=* visual analog scale.

### 2.8. Statistical analysis

Continuous variables were summarized as mean and standard deviation or median and interquartile range, and categorical variables were summarized as number and percentage. Between-group comparisons used t tests, Mann–Whitney *U* tests, chi-square tests, or Fisher exact tests as appropriate.^[7,12]^

The primary adjusted analysis used multivariable linear regression to estimate the association between combined-therapy and NDI change. Logistic regression was used for clinical response and recurrence. Propensity-score weighting was used as a sensitivity analysis to assess robustness to measured confounding.^[12,14]^ Two-sided *P* values below .05 were considered statistically significant, and effect estimates with confidence intervals were emphasized.^[7,12]^

## 3. Results

### 3.1. Cohort assembly and treatment exposure

Among 156 screened records, 30 were excluded because of nonradicular pain, prior cervical surgery, incomplete outcomes, or inability to complete treatment, leaving 126 records in the analytic cohort (Fig. 1A). Sixty patients received acupuncture alone and 66 received acupuncture-plus therapeutic massage (Fig. 1A). Monthly treatment-volume varied over the study period, and combined-therapy was more frequently documented in later months (Fig. 1B). The most frequent dominant symptomatic levels were C5–C6 and C6–C7, and the highest missingness rates were observed for 3-month follow-up and cervical range-of-motion measures (Figs. 1C–D). These data were used for the planned retrospective cohort analysis, although follow-up completeness remained important for interpreting recurrence and durability outcomes.

### 3.2. Baseline characteristics

Demographic and clinical characteristics were generally comparable between treatment groups (Table 1). The mean age was 50.8 years, 54.0% of patients were female, and median symptom duration was 6.5 weeks. Baseline VAS pain and NDI scores were similar between groups, and the distributions of disc herniation and spondylotic foraminal stenosis were balanced (Table 1). Overall, the cohorts appeared clinically comparable at treatment initiation, although unmeasured differences in patient preference, clinician judgment, and attendance capacity may have remained.

### 3.3. Treatment dose and adherence

The combined-therapy group completed a median of 12 total treatment visits compared with 10 visits in the acupuncture alone group (Table 2). The median number of massage sessions in the combined group was 10, showing that massage was delivered as an adjunct to acupuncture rather than as a replacement. Home exercise advice and posture or ergonomic education were documented for most patients in both groups (Table 2). These findings show that acupuncture-plus massage was commonly delivered with reasonable adherence in this outpatient setting.

**Table 2:** Treatment exposure, co-interventions, and adherence.

Exposure or care process	Overall (n = 126)	Acupuncture alone (n = 60)	Acupuncture + massage (n = 66)	*P*-value
Total treatment visits, median (IQR)	11 (9–14)	10 (8–12)	12 (10–15)	<.001
Acupuncture sessions completed, median (IQR)	10 (8–12)	10 (8–12)	12 (10–14)	.002
Massage sessions completed, median (IQR)	0 (0–10)	0 (0–0)	10 (8–12)	<.001
Completed >=80% scheduled sessions, n (%)	105 (83.3)	48 (80.0)	57 (86.4)	.34
Home exercise advice documented, n (%)	107 (84.9)	50 (83.3)	57 (86.4)	.63
Posture/ergonomic education documented, n (%)	99 (78.6)	46 (76.7)	53 (80.3)	.62
Analgesic use at end of treatment, n (%)	38 (30.2)	23 (38.3)	15 (22.7)	.06
3-mo follow-up available, n (%)	109 (86.5)	50 (83.3)	59 (89.4)	.31

IQR = interquartile range.

### 3.4. Pain, disability, and mobility outcomes

**Figure 2.**
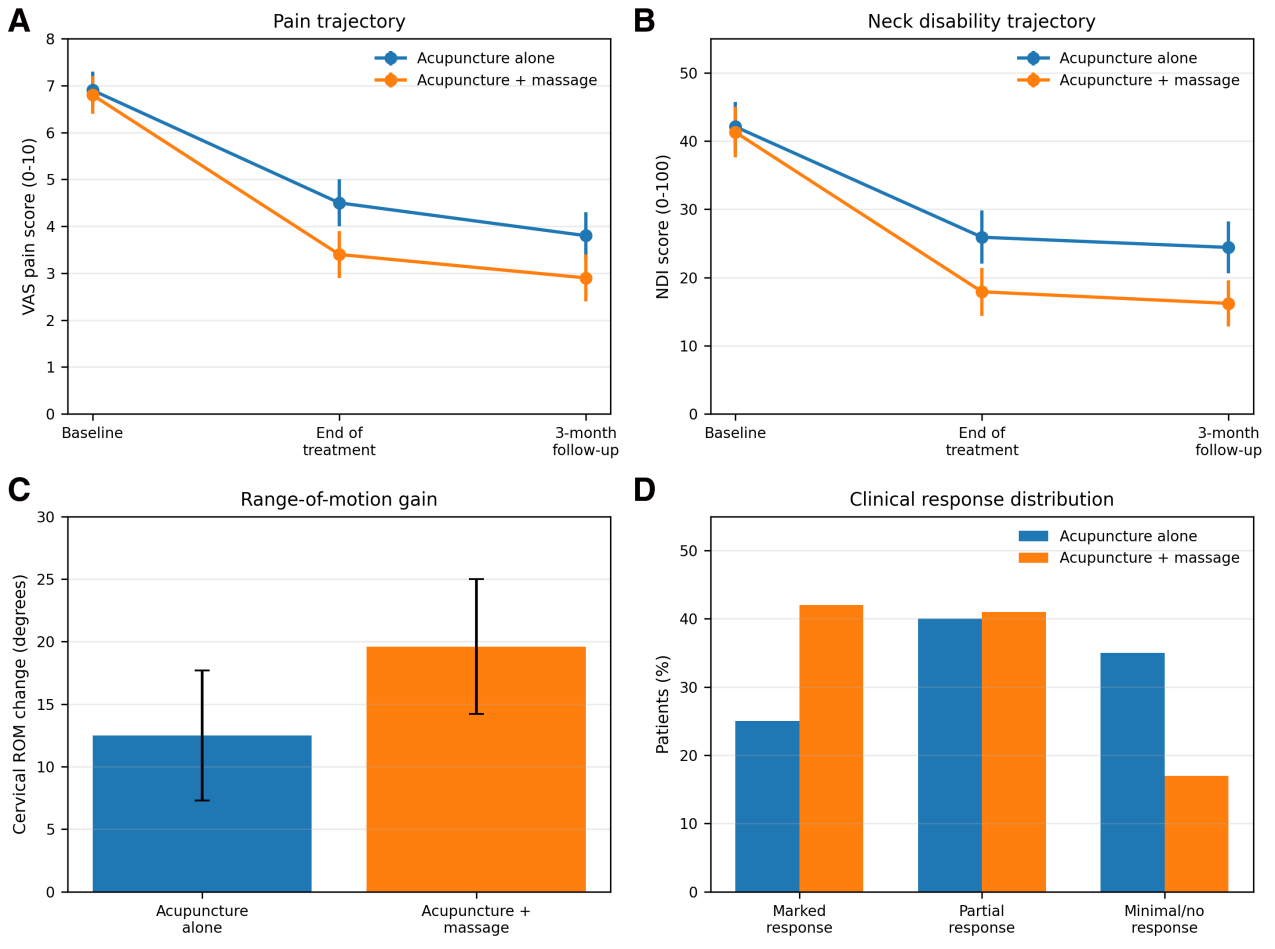
Pain, disability, mobility, and clinical response outcomes. (A) shows VAS pain trajectories; (B) shows NDI trajectories; (C) shows cervical range-of-motion gain; (D) shows clinical response distribution.

Both groups improved from baseline to the end of treatment, with larger gains in the acupuncture-plus-massage group (Table 3; Figure 2A-D). Compared with acupuncture alone, combined-therapy was associated with greater mean NDI reduction (−23.4 vs −16.2 points), greater VAS pain reduction (−3.4 vs −2.4 points), greater cervical range-of-motion gain (+19.6 vs +12.5 degrees), and a higher marked or partial clinical response rate (83.3% vs 65.0%) (Table 3; Figure 2A-D). These findings suggest that combining acupuncture with massage may be associated with greater observed short-term improvement in pain, disability, and cervical mobility.

**Table 3:** Clinical outcomes from baseline to end of treatment.

Outcome	Acupuncture baseline	Acupuncture end	Acupuncture change	Combined baseline	Combined end	Combined change	*P* for change
NDI score (0–100)	42.1 (10.8)	25.9 (11.6)	−16.2 (10.1)	41.3 (11.1)	17.9 (10.4)	−23.4 (11.0)	.002
VAS pain score (0–10)	6.9 (1.2)	4.5 (1.6)	−2.4 (1.5)	6.8 (1.1)	3.4 (1.5)	−3.4 (1.6)	.001
Cervical ROM, degrees	143.4 (27.1)	155.9 (28.4)	+12.5 (15.6)	141.9 (26.7)	161.5 (27.8)	+19.6 (16.2)	.014
Arm numbness score (0–10)	5.6 (1.7)	3.5 (1.8)	−2.1 (1.7)	5.5 (1.6)	2.6 (1.7)	−2.9 (1.8)	.010
Medication reduction, n/N (%)	18/60 (30.0)	−	−	31/66 (47.0)	−	−	.049
Marked/partial clinical response, n/N (%)	39/60 (65.0)	−	−	55/66 (83.3)	−	−	.018

Values are mean (SD) unless otherwise specified.

ND = neck disability index; ROM = range of motion; VAS = visual analog scale.

### 3.5. Adjusted associations and subgroup patterns

**Figure 3.**
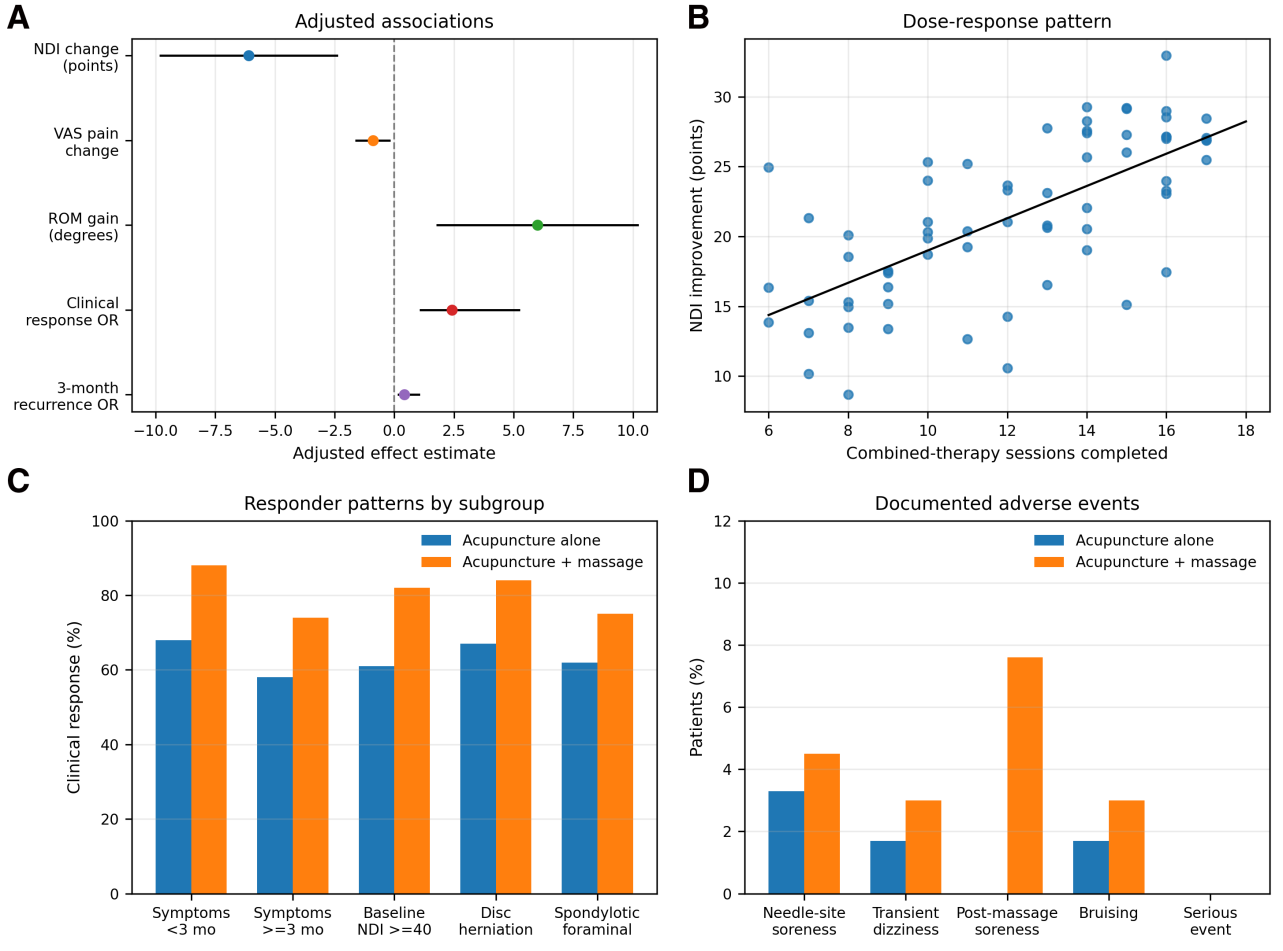
Adjusted associations, dose-response pattern, responder subgroups, and adverse events. (A) shows adjusted outcome estimates; (B) shows completed combined-therapy sessions vs NDI improvement; (C) shows clinical response by subgroup; (3) shows documented adverse events.

After adjustment for baseline severity and clinical covariates, combined-therapy remained associated with greater NDI improvement, greater VAS pain reduction, larger cervical range-of-motion gain, and higher odds of clinical response (Table 4; Figure 3A). Within the combined-therapy group, the number of completed sessions was positively associated with the magnitude of NDI improvement (Fig. 3B). Patients with symptom duration under 3 months, baseline NDI of 40 or higher, and disc herniation patterns showed larger response differences, although subgroup estimates were imprecise (Table 4; Figure 3C). These findings suggest that the observed association may reflect differences in treatment exposure, although this finding should be interpreted cautiously because treatment intensity was not randomized.

**Table 4:** Adjusted associations and subgroup findings favoring combined-therapy.

Outcome or subgroup	Effect scale	Adjusted estimate	95% CI	*P*-value
Primary: NDI change	Adjusted mean difference	−6.1 points	−9.8 to −2.4	.001
VAS pain change	Adjusted mean difference	−0.9 points	−1.6 to −0.2	.012
Cervical ROM gain	Adjusted mean difference	+6.0 degrees	1.8 to 10.2	.006
Marked/partial clinical response	Adjusted odds ratio	2.41	1.11 to 5.23	.026
3-mo recurrence	Adjusted odds ratio	0.42	0.17 to 1.03	.058
Symptoms <3 mo: clinical response	Adjusted odds ratio	3.02	1.10 to 8.31	.032
Baseline NDI >=40: clinical response	Adjusted odds ratio	2.86	1.01 to 8.08	.048
Disc herniation pattern: clinical response	Adjusted odds ratio	2.73	0.93 to 8.02	.067

Models adjusted for age, sex, symptom duration, baseline NDI, baseline VAS pain, imaging pattern, analgesic use, and visit number.

CI = confidence interval, NDI = neck disability index, ROM = range of motion, VAS = visual analog scale.

### 3.6. Recurrence and safety

Among patients with follow-up data, recurrent or persistent radicular symptoms at 3 months were documented in 10.2% of the combined-therapy group and 22.0% of the acupuncture alone group (Table 5). No serious treatment-related adverse-event was documented. Mild adverse-events included needle-site soreness, transient dizziness, post-massage soreness, and bruising (Table 5; Figure 3D). The primary NDI finding was consistent in propensity-weighted and multiple-imputation sensitivity analyses (Table 5). These findings suggest that combined-therapy had an acceptable safety profile in this cohort and showed a numerically lower recurrence rate, although this finding should be interpreted cautiously.

**Table 5:** Recurrence, safety events, and sensitivity analyses.

Outcome	Acupuncture alone (n = 60)	Acupuncture + massage (n = 66)	Effect estimate	*P*-value
3-mo recurrence among patients with follow-up, n/N (%)	11/50 (22.0)	6/59 (10.2)	OR 0.42 (0.14–1.23)	.11
Needle-site soreness, n (%)	2 (3.3)	3 (4.5)	Risk difference 1.2%	.72
Transient dizziness, n (%)	1 (1.7)	2 (3.0)	Risk difference 1.3%	.63
Post-massage soreness, n (%)	0 (0.0)	5 (7.6)	Risk difference 7.6%	.03
Bruising, n (%)	1 (1.7)	2 (3.0)	Risk difference 1.3%	.63
Serious treatment-related adverse event, n (%)	0 (0.0)	0 (0.0)	Not estimable	1.00
Propensity-weighted NDI change estimate	Reference	−5.7 points	95% CI −9.2 to −2.2	.002
Multiple-imputation NDI change estimate	Reference	−5.9 points	95% CI −9.5 to −2.3	.001

CI *=* confidence interval, NDI *=* neck disability index, OR *=* odds ratio.

## 4. Discussion

This retrospective cohort study used existing clinical records to evaluate the association between acupuncture-plus therapeutic massage and recovery in patients with cervical radiculopathy. Combined-therapy was associated with larger improvements in disability, pain, cervical mobility, and clinical response, with a numerically lower 3-month recurrence rate (Tables 3–5; Figure 2A-D; Figure 3A-D).

These findings are consistent with systematic reviews suggesting that acupuncture may improve pain outcomes in cervical spondylotic radiculopathy and that Tuina or massage may be useful as part of conservative multimodal care. However, the certainty of evidence remains limited by trial heterogeneity and risk of bias.^[3–6]^ The results are clinically plausible because massage may reduce soft-tissue guarding, local tenderness, and movement restriction, whereas acupuncture may contribute to pain modulation and reduction of radicular symptom intensity.^[3–5]^

Clinically, combined-therapy may provide a practical nonoperative option for selected patients without myelopathy, progressive motor deficit, infection, tumor, fracture, or other urgent surgical indications.^[1,2]^ The subgroup results suggest that patients with moderate to severe baseline disability, shorter symptom duration, and tolerable soft-tissue sensitivity may be a subgroup for further prospective evaluation of structured combined care.^[1,2,6]^

This study has several limitations. First, selection bias is possible because patients with incomplete outcomes, inability to complete treatment, or prior cervical surgery were excluded, and these exclusions may have been associated with prognosis (Fig. 1A).^[7,12]^ Second, missing data were present for follow-up and range-of-motion outcomes; missingness may have reflected improvement, poor response, or limited follow-up access rather than occurring completely at random (Fig. 1D).^[7,13]^

Third, confounding by indication remains a major limitation because assignment to massage was not randomized and may have been influenced by muscle spasm, patient preference, attendance capacity, clinician expectations, or differences in baseline prognosis.^[12,14]^ Fourth, adverse events were identified from routine documentation, so minor events may have been under-reported.^[7,12]^ Fifth, the single-center design limits generalizability to other practitioners, acupuncture protocols, massage techniques, visit schedules, and health systems.^[7,12]^

This study has several strengths. First, it used real-world clinical records to evaluate a commonly used conservative treatment strategy in routine outpatient practice. Second, the study directly compared acupuncture alone with acupuncture combined with therapeutic massage, allowing comparison of outcomes associated with acupuncture alone vs acupuncture combined with therapeutic massage. Third, the analysis included multiple clinically relevant outcomes, including neck disability, pain intensity, cervical ROM, clinical response, medication reduction, recurrence, and adverse events. Fourth, adjusted regression models and propensity-score sensitivity analysis were used to reduce the influence of measured baseline differences between groups. Finally, the inclusion of 3-month recurrence and safety data provides additional information beyond immediate symptom improvement.

Future research should prospectively validate these findings using standardized treatment dose, predefined co-intervention documentation, blinded or independent outcome assessment when feasible, longer follow-up of at least 6 to 12 months, and multicenter recruitment. A pragmatic prospective cohort study or randomized trial would help determine whether the observed associations represent a causal treatment effect and whether combined-therapy improves durable function, recurrence, medication use, and cost-effectiveness.^[7,12,14]^

## 5. Conclusion

Among patients with cervical radiculopathy, acupuncture combined with therapeutic massage was associated with greater observed short-term improvement in pain, neck disability, cervical ROM, and clinical response than acupuncture alone. These observational findings support further evaluation of coordinated multimodal conservative care.

## Author contributions

**Conceptualization:** Liqing Qi, Hong Liang, Zhan Wen Li.

**Data curation:** Liqing Qi, Hong Liang, Zhan Wen Li.

**Formal analysis:** Liqing Qi.

**Funding acquisition:** Liqing Qi, Zhan Wen Li.

**Investigation:** Liqing Qi, Zhan Wen Li.

**Project administration:** Liqing Qi, Hong Liang, Zhan Wen Li.

**Software:** Liqing Qi, Zhan Wen Li.

**Methodology:** Hong Liang, Zhan Wen Li.

**Supervision:** Hong Liang, Zhan Wen Li.

**Resources:** Zhan Wen Li.

**Validation:** Zhan Wen Li.

**Visualization:** Zhan Wen Li.

**Writing – original draft:** Liqing Qi, Hong Liang, Zhan Wen Li.

**Writing – review & editing:** Liqing Qi, Hong Liang, Zhan Wen Li.
